# A Novel Loosely Coupled Collaborative Localization Method Utilizing Integrated IMU-Aided Cameras for Multiple Autonomous Robots

**DOI:** 10.3390/s25103086

**Published:** 2025-05-13

**Authors:** Cheng Liu, Tao Wang, Zhi Li, Shu Li, Peng Tian

**Affiliations:** 1 State Key Laboratory of Explosion Science and Safety Protection, Beijing Institute of Technology, Beijing 100081, China; 3220235030@bit.edu.cn (C.L.); wang_tao@bit.edu.cn (T.W.); 2 Chongqing Innovation Center, Beijing Institute of Technology, Chongqing 401100, China; 3 School of Electrical Engineering, Liaoning University of Technology, Jinzhou 121000, China; 4 School of Information Science and Engineering, Chongqing Jiaotong University, Chongqing 401100, China; tianpeng@cqjtu.edu.cn

**Keywords:** loosely coupled autonomous localization, visual–inertial odometry, multiple robots collaborative localization, data fusion

## Abstract

IMUs (inertial measurement units) and cameras are popular sensors for autonomous localization due to their convenient integration. This article proposes a collaborative localization method, the CICEKF (collaborative IMU-aided camera extended Kalman filter), with a loosely coupled and two-step structure for the autonomous locomotion estimation of collaborative robots. The first step is for single-robot localization estimation, fusing and connecting the IMU and visual measurement data on the velocity level, which can improve the robustness and adaptability of different visual measurement approaches without redesigning the visual optimization process. The second step is for estimating the relative configuration of multiple robots, which further fuses the individual motion information to estimate the relative translation and rotation reliably. The simulation and experiment demonstrate that both steps of the filter are capable of accomplishing locomotion estimation missions, standalone or collaboratively.

## 1. Introduction

### 1.1. Motivation

The autonomous localization of multiple robots working on ground and aerial space is well-explored and is achieved with different sensor setups such as visual measurement [1], GNSS (global navigation satellite system) [2], and visual–inertial equipment [3]. However, for search and rescue robots facing emergent or indoor situations [4,5], collaborative autonomous navigation under unknown and GNSS-denied environments [6] aiming for group closed-loop motion control [7,8] is still challenging due to the need for accuracy against environmental uncertainty. Visual–inertial approaches are popular for their robustness and real-time performance against interferences [9,10] to solve localization problems for both individual and group robots. Although the vision-based odometry and SLAM (simultaneous localization and mapping) methods have achieved great accuracy for their batch-optimization capability [11,12,13], the vision-only approaches are genuinely weak in solving the collaborative localization problem owing to the lack of a common world coordinate frame as an anchorage [14,15]. On the other hand, IMUs have good real-time responsiveness, while the inherent drift affects the long-term performance. 

Therefore, it is necessary to explore approaches with an open and flexible data fusion framework to improve the accuracy of cooperative group localization, which can balance the instantaneity of the IMU sensors and the accuracy of visual measurement for collaborative agents under GNSS-restricted situations.

### 1.2. Related Work

The research on autonomous localization based on the data fusion of the IMU and visual measurement mainly focuses on tightly and loosely coupled approaches. 

The tightly coupled methods are typically biased towards visual measurement when integrating the IMU’s real-time output from the gyroscope and accelerometer into the batch-optimization process. The state-of-the-art single-robot visual–inertial localization methods, such as ORB-SLAM v3 [16] and MSCKF 2.0 [17], have shown great robustness and accuracy. TH Nguyena et al. [18] proposed a two-step optimization-based collaborative visual–inertial range localization method that separately estimates the relative transformation and single-robot localization. Tian et al. [19] proposed a distributed multi-robot system for robust and dense metric semantic SLAM that detects loop closures and performs distributed trajectory estimation when communication among individuals is available. Though achieving great performance in the tests with the benefits of post-optimization, such tightly coupled methods usually lack both openness due to the fixed optimization structure and low real-time performance due to the high computing power demand. These result in inefficiency in dealing with uncertain topology and different mounted sensors of multi-agents. 

Loosely coupled methods are naturally fit to perform relative localization due to their flexible algorithm structure and timeliness by emphasizing the IMU update [20]. The main weakness is their unsatisfactory accuracy owing to the inevitable drift of the IMU measurement, which should be carefully handled. 

Kelly and Sukhatme [21] achieved the self-calibration of a monocular visual–inertial system with a specifically designed UKF (unscented Kalman filter). Following a similar approach, Weiss and Siegwart [22] introduced the estimation of the world coordinate frame drift of the visual measurement into a multi-level EKF (extended Kalman filter) for a better trajectory estimation for a single drone. Based on further research, Achtelik and Weiss [23] achieved the estimation of the relative configuration of two drones by utilizing a multi-level EKF to handle the loosely coupled visual–inertial data fusion. Researchers have introduced new filter techniques, such as the invariant EKF based on Lie algebra [24], the multi-state constraint Kalman filter [25], and the equivariant filter [26,27], into the trajectory estimation with visual–inertial equipment. 

These loosely coupled methods usually treat visual measurement as a black box, thus achieving a flexible structure with great portability across different sensor setups. However, owing to only considering the update with IMU data on the acceleration and velocity levels, the final correction on the position level with the visual measurement result may lose accuracy when a sudden visual measurement instability is encountered.

### 1.3. Our Approach

This article presents a loosely coupled filter-based approach, the CICEKF, with two steps aiming for both the autonomous localization of a single robot and the collaborative estimation of relative multi-robot localization. The time complexity of the method is $O\left(n\right)$. The SR-CICEKF (single-robot CICEKF) step focuses on the fusion of the data on the stereo vision and IMU velocity levels from the integrated sensors for a robust locomotive estimation. The MR-CICEKF (multi-robot CICEKF) step fuses outcomes of the SR-CICEKF from multiple robots to estimate the real-time relative position and attitude from each slave robot to the master robot. The two steps can run separately as standalone filters or serially as a complete CICEKF. Furthermore, to demonstrate the feasibility of the CICEKF, the observability is proven.

This article is organized as follows: The design of the state vectors of the CICEKF is described in Section 2, a detailed description of the propagation and update processes of the filter is provided in Section 3, the local weak observability of the CICEKF is demonstrated in Section 4, simulation with quintic curves is presented in the Section 5, and real-world experiments were carried out and are analyzed in Section 6. The conclusions of this study are provided in Section 7.

## 2. Design of the State Vector

### 2.1. Definition of Variables

**Figure 1:**
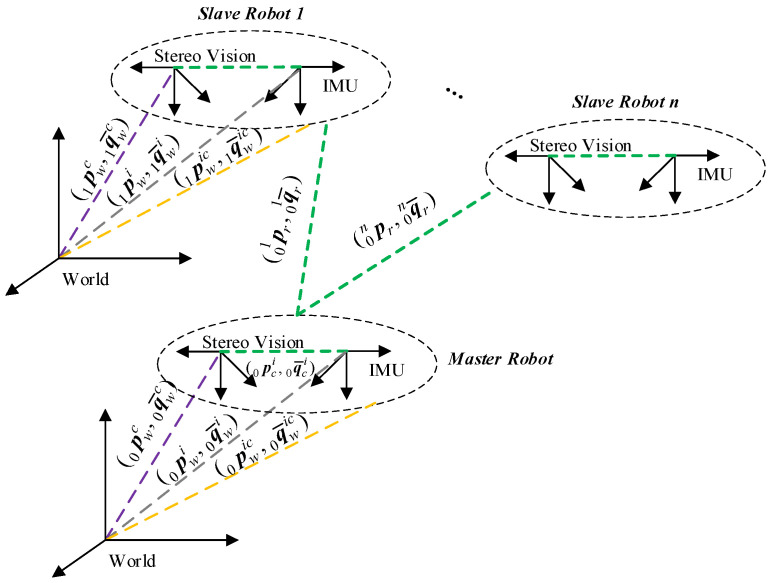
Relationship of multi-robot coordinate frames.

To focus the discussion on the filter-based data fusion process, the stereo vision is treated as black-box autonomous locomotion measurement equipment. It is assumed that each robot is equipped with an IMU-aided stereo-vision sensor suite, of which the relative position and attitude of the IMU and the cameras are already calibrated without further drifting. The locomotive variables and relative transformations are defined as shown in Figure 1, and the coordinate frames and the notation of variables used in this study are provided in Table 1. 

The fixed index of the master robot is set as 0, and the indexes of the slave robots are numbered from 1 to n.pcin and q¯cin represent the calibrated relative position and attitude, respectively, between the IMU and the stereo-vision coordinate frames of the n-th robot. The n-th robot’s linear translation pwcn and the rotation q¯wcn obtained from the black-box stereo vision are measured by referring to the world coordinate frame. pwin and q¯win are the linear translation and the rotation of the IMU inspected in the world coordinate frame, respectively. The coupled position and attitude fusion results of each IMU–camera suite, i.e., pwicn and q¯wicn, are attached to its IMU coordinate frame to simplify the filtering process. The raw measurement outputs of the n-th IMU are the linear acceleration amin and angular velocity ωmin, which are measured in its body-following coordinate frame. The relative configuration consists of the relative position and attitude between the n-th slave robot and the master robot, which are pr0n and q¯r0n, respectively. The units used in this article are described in the SI. This design of the master/slave relationship among multiple agents allows for convenient modification of the topology under practical situations.

In this study, the drift of the vision’s world coordinate frame is not considered since it is slow enough to be treated as noise. Furthermore, all noise variables are modeled as a random walk·. The small-angle assumption is applied, which means letting the rotation angle be converted from $\bar{q}$ be θ, and the error of $\bar{q}$ is written as $\delta \bar{q}={\left[q_{0},\delta q^{T}\right]}^{T}\approx {\left[1,\frac{1}{2}\delta \theta^{T}\right]}^{T}$ when $\theta \ll 1^{\circ }$ [28].

### 2.2. Construction of the SR-CICEKF State Vector

From this point onwards, for the filter construction considering the IMU-aided stereo-vision suite of any *single* robot, the left corner markers are omitted for simplification, such as pwi0=pwi. 

Thus, the vector with 29 elements representing the state of the SR-CICEKF is defined as follows:

(1) X=pwicT vwcT vwiT q¯wicT q¯wiT ωccT ωiiT baiT bωiTT,

where $v_{w}^{c}$ is the derivative of $p_{w}^{c}$; $v_{w}^{i}$ is integrated from $a_{mi}$, which possesses a bias $b_{ai}$; $\omega_{c}^{c}$ is the angular velocity of the stereo vision body, which is inspected in the IMU coordinate frame rather than the vision coordinate frame; and $\omega_{i}^{i}$ is the angular velocity of the IMU body in its own coordinate frame with a bias $b_{\omega i}$. Additionally, there are ${\dot{v}}_{w}^{i}=a_{w}^{i}-g=R_{w}^{i}\left(a_{mi}-b_{ai}-n_{a_{mi}}\right)-g$ and $\omega_{i}^{i}=\omega_{mi}-b_{\omega i}-n_{\omega_{mi}}$, where $n_{a_{mi}}$ and $n_{\omega_{mi}}$ are the measurement noises, and g is the gravity vector with respect to the world coordinate frame.

#### 2.2.1. Coupling Process for the SR-CICEKF

The independent coupling coefficients of the linear and angular velocities are $\mu_{v}\in [0,1]$ and $\mu_{\omega }\in [0,1]$, respectively, ensuring adjustability while enhancing motion estimation accuracy without affecting observability.

The linear velocity coupling is straightforward and written as follows:

(2) $$v_{w}^{ic}=\mu_{v}v_{w}^{i}+(1-\mu_{v})R_{c}^{i}v_{w}^{c}.$$

The coupling process of angular velocities cannot be conducted directly since the rotations of the IMU and vision body are inspected in different coordinate frames. Assuming ${\bar{q}}_{w}^{ic}={\bar{q}}_{w}^{c^{\prime }}{\bar{q}}_{w}^{i}$, the following equations are obtained referring to [29]:

(3) $$\frac{d}{dt}{\left({\bar{q}}_{w}^{i}\right)}^{\mu_{\omega }}=\frac{1}{2}{\left({\bar{q}}_{w}^{i}\right)}^{\mu_{\omega }}⊗\left(\mu_{\omega }{\bar{\omega }}_{i}^{i}\right),$$

(4) $$\frac{d}{dt}{\left({\bar{q}}_{w}^{c^{\prime }}\right)}^{1-\mu_{\omega }}=\frac{1}{2}{\left({\bar{q}}_{w}^{c^{\prime }}\right)}^{1-\mu_{\omega }}⊗\left(\left(1-\mu_{\omega }\right){\bar{\omega }}_{c}^{c}\right),$$

where ${\bar{q}}_{w}^{c^{\prime }}$ is achieved by inspecting the rotation of the stereo vision body in the IMU coordinate frame, and ${\bar{q}}^{\mu }$ is the μ-th power of $\bar{q}$, which denotes a unit quaternion scaling the rotation angle around the virtual axis with $\mu \in \left[0,1\right]$ [30]. 

According to Appendix A, Equations (3) and (4), ${\bar{q}}_{w}^{ic}$ can thus be derived as follows:

(5) $$\begin{array}{l} {\dot{\bar{q}}}_{w}^{ic}=\frac{d}{dt}{\left({\bar{q}}_{w}^{i}\right)}^{\mu_{\omega }}⊗{\left({\bar{q}}_{w}^{c^{\prime }}\right)}^{1-\mu_{\omega }}+{\left({\bar{q}}_{w}^{i}\right)}^{\mu_{\omega }}⊗\frac{d}{dt}{\left({\bar{q}}_{w}^{c^{\prime }}\right)}^{1-\mu_{\omega }}\\ =\frac{\mu_{\omega }}{2}{\left({\bar{q}}_{w}^{i}\right)}^{\mu_{\omega }}⊗{\bar{\omega }}_{i}^{i}⊗{\left({\bar{q}}_{w}^{c^{\prime }}\right)}^{1-\mu_{\omega }}+\frac{1-\mu_{\omega }}{2}{\bar{q}}_{w}^{ic}⊗{\bar{\omega }}_{c}^{c} \end{array},$$

where $\bar{\omega }=[0,\omega ]$.

#### 2.2.2. Simplification of the SR-CICEKF State Vector

To simplify the further discussion, the SR-CICEKF state vector in Equation (1) is rewritten as follows:

(6) X=pic Tvc Tvi Tq¯ic Tq¯iωcTωiT TbaTbωTT.

The corresponding derivatives are as follows:

(7) p˙ic=vic=μvvi+(1−μv)Rcivc, v˙c=nvc, v˙i=ai−g=Riami−bai−nami−g, q¯˙ic=μω2q¯i⊗μωω¯i⊗q¯c+1-μω1-μω2q¯ic⊗ω¯c, q¯˙i=12q¯i⊗ω¯i, ω˙c=nωc, ω˙i=ddtωmi−bωi−nωmi, b˙ai=nbai, b˙ωi=nbωi,

where ${\bar{q}}_{c}={\bar{q}}_{w}^{c^{\prime }}{\bar{q}}_{c}^{i}⊗{\bar{q}}_{w}^{c}$.

#### 2.2.3. Error of the SR-CICEKF State Vector

The error state contributes to the propagation matrices and is essential for the filtering process. $\tilde{X}$ contains 27 elements, written as follows:

(8) X˜=Δpic TΔvc TΔvi Tδθic Tδθi TΔωc TΔωi TΔbaiΔTbωiTT.

The update rate of this filter-based approach is determined by the IMU refresh rate. Thus, small high-order terms in the process can be omitted, such as δq⋅Δω, δq⋅δq, and δq⋅n since the IMU can usually run faster than 100 Hz.

To build the connection from the state vector to the propagation process, the derivative form of $\tilde{X}$ should be inspected. 

The derivative of the coupled translation error can be obtained directly as follows:

(9) $$\dot{\Delta p_{ic}}=\mu_{v}\Delta v_{i}+(1-\mu_{v})R_{c}^{i}\Delta v_{c}.$$

Let ${\hat{a}}_{i}=a_{mi}-{\hat{b}}_{ai}$ and $\Delta b_{ai}=b_{ai}-{\hat{b}}_{ai}$, and according to $R_{i}=R\left({\bar{q}}_{i}\right)\approx {\hat{R}}_{i}\left(I_{3}+\left\lfloor \delta \theta_{i}\times \right\rfloor \right)$ achieved by following Appendix A, the equations are as follows:

(10) $$\begin{array}{l} \dot{\Delta v_{i}}=a_{i}-{\hat{a}}_{i}=R_{i}(a_{mi}-b_{ai}-n_{a_{mi}})-g-{\hat{R}}_{i}(a_{mi}-{\hat{b}}_{ai})+g\\ \approx -{\hat{R}}_{i}\left\lfloor {\hat{a}}_{i}\times \right\rfloor \delta \theta_{i}-{\hat{R}}_{i}\Delta b_{ai}-n_{a_{mi}} \end{array},$$

where the higher-order terms are omitted.

According to Appendix A, the equations are as follows:

(11) q¯ic*=q¯c1−μω*⊗q¯iμω*q¯iμω*⊗q¯iμω=1q¯^c1−μω*⊗p¯ic⊗q¯^c≙1−μωR^q¯^c1−μωTpic=R^μωcpicT.

By subjecting Equation (11) to Equation (5), there is obtained as follows:

(12) δq¯ic.≈−μω202Rμωcω^iT×δqic−0q0RμωcΔTωi−1-μω202ω^c×δqic−0q0Δωc,

where $\Delta \bar{\omega }=\bar{\omega }-\hat{\bar{\omega }}$ is applied, and the high-order terms are disregarded. 

Following the small-angle assumption, let $\delta {\bar{q}}_{ic}={\left[q_{0},\delta {q_{ic}}^{T}\right]}^{T}\approx {\left[1,\frac{1}{2}\delta \theta_{ic}^{T}\right]}^{T}$, and there is as follows:

(13) δθic.=−μωRμωcω^iT×δθic−RμωcΔTωi−1−μωω^c×δθic−Δωc.

Similarly, there is as follows:

(14) $$\dot{\delta {\bar{q}}_{i}}\approx \left[\begin{array}{l} 0\\ -\left\lfloor {\hat{\omega }}_{i}\times \right\rfloor \delta q_{i} \end{array}\right]-\left[\begin{array}{l} 0\\ \frac{1}{2}q_{i0}\Delta \omega_{i} \end{array}\right],$$

where ${\hat{\omega }}_{i}=\omega_{mi}-{\hat{b}}_{\omega i}$ and $\Delta b_{\omega i}=b_{\omega i}-{\hat{b}}_{\omega i}$ are applied. 

Following the small-angle assumption, let $\delta {\bar{q}}_{i}={\left[q_{0},\delta {q_{i}}^{T}\right]}^{T}\approx {\left[1,\frac{1}{2}\delta \theta_{i}^{T}\right]}^{T}$, and there is as follows:

(15) $$\dot{\delta \theta_{i}}=-\left\lfloor {\hat{\omega }}_{i}\times \right\rfloor \delta \theta_{i}-\Delta \omega_{i}=-\left\lfloor {\hat{\omega }}_{i}\times \right\rfloor \delta \theta_{i}-\Delta b_{\omega i}-n_{\omega_{mi}}.$$

For other terms in the error state, there is no change compared to the state vector since they are obtained directly from the measurement. Therefore, there is as follows:

(16) $${\dot{\Delta v}}_{c}=n_{v_{c}},\dot{\Delta \omega_{c}}=n_{\omega_{c}},\dot{\Delta \omega_{i}}=\frac{d}{dt}\left(\Delta b_{\omega i}+n_{\omega_{mi}}\right)=n_{\omega i},\dot{\Delta b_{ai}}=n_{b_{ai}},\dot{\Delta b_{\omega i}=}n_{b_{\omega i}}.$$

### 2.3. Construction of the MR-CICEKF State Vector

One primary purpose of this study is to achieve a relative configuration between two robots. Therefore, the IMU coordinate frame, which is inherent and fixed after calibration, is chosen as the anchorage to establish the filter. For the conditions with multiple robots running simultaneously, the relative configuration chain can be easily developed with a convenient adjustment to the topology.

The construction process of the MR-CICEKF state equations is partially similar to SR-CICEKF. The input of the MR-CICEKF is obtained from the output of SR-CICEKF, and the state vector with 19 elements is written as follows:

(17) Y=pr0 0Tvwi 1Tvwi 0Tq¯r 0Tωii 1TωiiTT,

where prT0 and q¯rT0 have been described previously, vwi0 and ωii0 are the IMU’s linear velocity and angular velocity obtained after applying the SR-CICEKF to the master robot’s sensor suite, respectively; and vwi1 and ωii1 are the outputs of the slave robot.

The derivative form of Y connects the variables in the state vector. The derivative of the relative translation is obtained as follows:

(18) pwi1−0pwi=Rq¯r0⋅pr0⇔ddtpwi1−0pwi=Rr⋅ωii0×⋅p0+rRr⋅p˙r0⇔p˙r0=vwir−ωii0×⋅pr0,

where vwir=RrT⋅vwi1−vwi0 is the relative linear velocity of the robots.

According to q¯r0=0q¯wi*⊗1q¯wi, the derivative of the relative rotation is written as follows:

(19) q¯˙r0=ddtq¯wi*0⊗1q¯wi+0q¯wi*⊗ddtq¯wi1=12q¯r0⊗1ωii−0ωii⊗0q¯r.

#### 2.3.1. Simplification of the MR-CICEKF State Vector

To simplify the further discussion, the MR-CICEKF state vector is rewritten as follows:

(20) Y=pr Tvi0 Tvi1 Tq¯r Tωi0 Tωi1TT.

Additionally, there is vwir=vr. The derivatives of the relative motion variables are written as follows:

(21) $${\dot{p}}_{r}=v_{r}=R_{r}^{T}\cdot \left(v_{i1}-v_{i0}\right)-\left\lfloor \omega_{i0}\times \right\rfloor \cdot p_{r},\;{\dot{\bar{q}}}_{r}=\frac{1}{2}\left({\bar{q}}_{r}⊗\omega_{i1}-\omega_{i0}⊗{\bar{q}}_{r}\right),\;{\dot{v}}_{i0}=n_{v_{i0}},\;\;{\dot{v}}_{i1}=n_{v_{i1}},\;{\dot{\omega }}_{i0}=n_{\omega_{i0}},\;{\dot{\omega }}_{i1}=n_{\omega_{i1}}.$$

#### 2.3.2. Error of the MR-CICEKF State Vector

$\tilde{Y}$ contains 18 elements and is written as follows:

(22) Y˜=Δpr TΔvi0 TΔvi1 Tδθr TΔωi0 TΔωi1TT.

Following Appendix A, $R_{r}^{T}=\left(I_{3}-\left\lfloor \delta \theta_{r}\times \right\rfloor \right){{\hat{R}}_{r}}^{T}$ holds. According to $p_{r}={\hat{p}}_{r}+\Delta p_{r}$, by omitting high-order terms, there is as follows:

(23) Δp˙r=Rrvi1−vi0T−ωi0×pr−R^rv^i1−v^i0T−ω^i0×p^r≈R^rΔTvi1−R^rΔTvi0+R^rvi1−vi0T×δθr+p^r×Δωi0−ω^i0×Δpr,

where $\omega_{i0}={\hat{\omega }}_{i0}+\Delta \omega_{i0}$, $v_{i0}={\hat{v}}_{i0}+\Delta v_{i0}$ and $v_{i1}={\hat{v}}_{i1}+\Delta v_{i1}$ are applied.

Resembling the deduction process of Equations (11) and (12), the error of the relative rotation can be obtained as follows:

(24) δq¯˙r=0−ω^i1×δqr+012Δωi1−012R^rΔTωi0,

After applying the small-angle assumption, the result is as follows:

(25) δθ˙r=−ω^i1×δθr+Δωi1−R^rΔTωi0,

Since the linear and angular velocities are generated from the SR-CICEKF process, which can be seen as a measurement process, there is as follows:

(26) $$\Delta v_{i0}=n_{v_{i0}},\Delta v_{i1}=n_{v_{i1}},\Delta \omega_{i0}=n_{\omega_{i0}},\Delta \omega_{i1}=n_{\omega_{i1}}.$$

### 2.4. Relationships Among the Variables in the CICEKF

**Figure 2:**
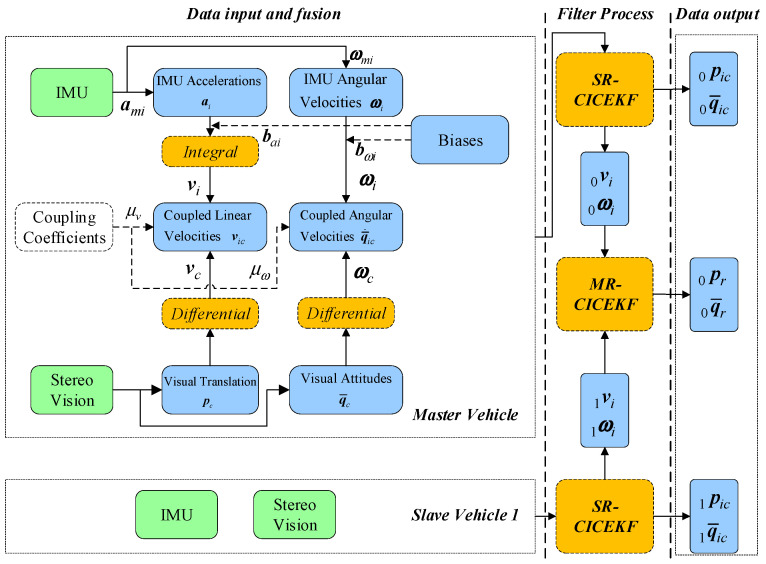
Relationships among the variables in the CICEKF state vector.

By analyzing the construction of the state vectors, it can be concluded that the SR-CICEKF and the MR-CICEKF are tightly connected in the inheritance of variables and the synchronous update, which are assembled as an entire CICEKF process. The relationships among the variables in the CICEKF states are shown in Figure 2. The whole filter is divided into three parts: data preparation, filter process, and data output. The data preparation contains the data input and fusion for both the master and slave robots as individuals. The filter process is separated into two steps, which are SR-CICEKF and MR-CICEKF.

## 3. Propagation and Update of the CICEKF

The propagation process is key to connecting different layers of the filter. The update process introduces measurement data to correct the entire data fusion process.

### 3.1. Propagation and Measurement of the SR-CICEKF

#### 3.1.1. Propagation of the SR-CICEKF

Let the continuous state transition matrix of the SR-CICEKF be $F_{c}$ and the continuous noise transition matrix be $G_{c}$, which are constant over each integration time step. By linearizing the continuous-time errors of a CICEKF state, there is as follows [31]:

(27) $$\dot{\tilde{X}}=F_{c}\tilde{X}+G_{c}n_{X},$$

where the state noise vector is $n_{X}={\left[n_{\omega_{c}}^{T}\;n_{a_{mi}}^{T}\;n_{\omega_{c}}^{T}\;n_{\omega_{mi}}^{T}\;n_{b_{ai}}^{T}\;n_{b_{\omega i}}^{T}\right]}^{T}$.

By applying Taylor’s formula, the discrete form of Equation (27) is as follows [31]:

(28) $$\left\{\begin{array}{l} F_{d}=\exp(F_{c}\Delta t)=I_{d}+F_{c}\Delta t+\frac{1}{2!}F_{c}^{2}\Delta t^{2}+\ldots \\ Q_{d}=\int_{t}^{t+\Delta t}F_{d}(\tau )G_{c}Q_{c}G_{c}^{T}F_{d}{\left(\tau \right)}^{T}d\tau \end{array}\right.,$$

where $F_{d}$ is the discrete form of $F_{c}$, and the continuous noise covariance matrix $Q_{c}=diag(\sigma_{n_{v_{c}}}^{2},\sigma_{n_{a_{i}}}^{2},\sigma_{n_{\omega_{c}}}^{2},\sigma_{n_{\omega_{i}}}^{2},\sigma_{n_{b_{ai}}}^{2},\sigma_{n_{b_{\omega i}}}^{2})$ is a diagonal matrix with $Q_{d}$ being its discrete form.

By considering the first-order expansion, after computing the Jacobians $\frac{\partial \dot{\tilde{X}}}{\partial \tilde{X}}$ to obtain $F_{c}$, the expression of $F_{d}$ can be written as follows:

(29) Fd=I3(1−μv)RciΔtμvI3Δt…03×1803I3…03×210303I303−R^ia^i×Δt0303−R^iΔt03030303I3A031−μωΔtI3μωRμωcΔTt030303030303I3−ω^i×Δt030303−ΔtI3012×15…I12×1227*27,

where A=I3−μωRμωcω^iT×+1−μωω^c×Δt.

Following Equations (28) and (29), $Q_{d}$ can be obtained from $G_{c}$, which is derived according to $\frac{\partial \dot{\tilde{X}}}{\partial n_{X}}$ and written as follows:

(30) $$G_{c}={\left[\begin{array}{cccccc} & & 0_{3\times 18} & & & \\ I_{3} & 0_{3} & 0_{3} & 0_{3} & 0_{3} & 0_{3}\\ 0_{3} & -{\hat{R}}_{i} & 0_{3} & 0_{3} & 0_{3} & 0_{3}\\ & & 0_{3\times 18} & & & \\ 0_{3} & 0_{3} & 0_{3} & -I_{3} & 0_{3} & 0_{3}\\ 0_{3} & 0_{3} & I_{3} & 0_{3} & 0_{3} & 0_{3}\\ 0_{3} & 0_{3} & 0_{3} & 0_{3} & I_{3} & 0_{3}\\ & & 0_{6\times 18} & & & \end{array}\right]}_{27*18}.$$

#### 3.1.2. Measurement of the SR-CICEKF 

For an autonomous robot, the keyframes of the visual measurement can possess high localization accuracy for its post-batch optimization with visual features. Let the measurement vector of the SR-CICEKF at the k-th step be $z_{k}$ with its error form as z˜k=z˜pTz¯˜qTz˜vTz˜ωTT, where ${\tilde{z}}_{P}$ is the error of the linear position, the quaternion ${\tilde{\bar{z}}}_{q}$ is the error of the rotation, ${\tilde{z}}_{v}$ is the error of the linear velocity, and ${\tilde{z}}_{\omega }$ is the error of the angular velocity. The measurement noise of the variables is simplified as nm=npTnθTnvTnωTT.

Following $\tilde{x}=x-\hat{x}$ and $\delta \bar{q}={\hat{\bar{q}}}^{*}⊗\bar{q}$, the entire description of ${\tilde{z}}_{P}$,${\tilde{\bar{z}}}_{q}$, and ${\tilde{z}}_{v}$ based on variables in the error state vector of the SR-CICEKF can be deducted as follows:

(31) z˜p=Δp+R^icpic×δθic+npz¯˜q=q¯^ic⊗*q¯ci⊗q¯c=δq¯ic≈112δθic+nθz˜v=−μvΔtR^ia^i×δθi−μvΔtR^iΔba+(1−μv)RciΔvc+nv.

In particular, the angular velocities of both sensors cannot be directly subtracted since they do not share the same coordinate frame. Therefore, ${\tilde{z}}_{\omega }$ is obtained indirectly from Equation (15) as follows:

(32) z˜ω=δθic.Δt+nω=−ΔtμωRμωcω^iT×+1−μωω^c×δθic+1−μωΔtΔωc+μωΔtRμωcΔTωi+nω.

The measurement matrix $H_{k}$ can be recovered from ${\tilde{z}}_{k}≃H_{k}{\tilde{X}}_{k}+n_{m}$, which is written as follows:

(33) $$H_{k}={\left[\begin{array}{ccccccc} I_{3\times 3} & & 0_{3\times 6} & & {\hat{R}}_{ic}\left\lfloor \left(p_{i}^{c}\right)\times \right\rfloor & & 0_{3\times 15}\\ 0_{3\times 9} & & & \frac{1}{2}I_{3\times 3} & & & 0_{3\times 15}\\ 0_{3\times 3} & \Delta t(1-\mu_{v})R_{c}^{i} & 0_{3\times 6} & -\mu_{v}\Delta t{\hat{R}}_{i}\left\lfloor {\hat{a}}_{i}\times \right\rfloor & 0_{3\times 6} & -\mu_{v}\Delta t{\hat{R}}_{i} & 0_{3\times 3}\\ 0_{3\times 9} & B & 0_{3\times 3} & \left(1-\mu_{\omega }\right)\Delta tI_{3\times 3} & \mu_{\omega }\Delta t{R_{\mu_{\omega }c}}^{T} & & 0_{3\times 6} \end{array}\right]}_{12*27},$$

where B=−ΔtμωRμωcω^iT×+1−μωω^c×.

### 3.2. Propagation and Measurement of the MR-CICEKF 

The processes of the propagation and measurement of the MR-CICEKF are similar to those of the SR-CICEKF and focus on relative motion estimation.

Following the deduction of the propagation matrix from Equation (27) to (28), let the noise vector of the state variables of the MR-CICEKF be $n_{r}={\left[{n_{v_{i0}}}^{T}{n_{v_{i1}}}^{T}{n_{\omega_{i0}}}^{T}{n_{\omega_{i1}}}^{T}\right]}^{T}$. And the discrete relative state transition matrix is written as follows:

(34) Fdr=I3−ω^i0×−R^rTR^rTR^rvi1−vi0T×p^r×0303I3…03×120303I3…03×903×9…I3−ω^i1×−R^rTI303×12…I30303×15…I318×18.

The discrete noise covariance matrix is Qdr=∫tt+ΔtFdr(τ)GcrQcrGcrFdrT(τ)Tdτ, where there is as follows:

(35) $$G_{c}^{r}={\left[\begin{array}{llll} 0_{3} & 0_{3} & {\;0}_{3} & {\;0}_{3}\\ I_{3} & 0_{3} & {\;0}_{3} & {\;0}_{3}\\ 0_{3} & I_{3} & {\;0}_{3} & {\;0}_{3}\\ 0_{3} & 0_{3} & {\;0}_{3} & {\;0}_{3}\\ 0_{3} & 0_{3} & I_{3} & {\;0}_{3}\\ 0_{3} & 0_{3} & {\;0}_{3} & I_{3} \end{array}\right]}_{18\times 12}.$$

By defining the measurement noise vector of the MR-CICEKF as nrm=nrpTnrθTT, let the measurement error of the MR-CICEKF at the k-th step be z˜rk=z˜rpTz˜rqTT, then, there is as follows:

(36) z˜rp=pc1−pc0−p^rz˜rq=δq¯r=q¯c*0⊗q¯c1⊗q¯^r≈112⋅δθr+nrθ.

After inspecting the relationship between the measurement and the desired output of the filter through ${\tilde{z}}_{k}^{r}≃H_{k}^{r}{\tilde{Y}}_{k}+n_{rm}$, the measurement matrix $H_{k}^{r}$ can be deducted as follows:

(37) $$H_{k}^{r}={\left[\begin{array}{cccccc} I_{3\times 3} & 0_{3\times 3} & 0_{3\times 3} & 0_{3\times 3} & 0_{3\times 3} & 0_{3\times 3}\\ 0_{3\times 3} & 0_{3\times 3} & 0_{3\times 3} & \frac{1}{2}I_{3\times 3} & 0_{3\times 3} & 0_{3\times 3} \end{array}\right]}_{6\times 18}.$$

### 3.3. Entire CICEKF Process

From the analysis above, it can be concluded that the CICEKF has two major EKF-based loops to update and correct the relevant states. The first loop, i.e., the SR-CICEKF, is shown in Algorithm 1, and the second loop, i.e., the MR-CICEKF, is shown in Algorithm 2, where $R_{k}$ and $R_{k}^{r}$ are the measurement noise matrices pre-established as described in [32,33], $K_{k}$ and $K_{k}^{r}$ are the Kalman gains, $P_{k+1|k}$ and $P_{k+1|k}^{r}$ are the prior covariance matrices of error, and $P_{k+1|k+1}$ and $P_{k+1|k+1}^{r}$ are the posterior covariance matrices of the error.

The first loop governs the propagation, measurement, and update of a single robot’s motion state by fusing the measurement data of the stereo vision and the IMU on the velocity level. The second loop is focused on the estimation of the relative positions and rotations of multiple slave robots with respect to the master robot. This dual-loop design keeps the independence of either algorithm for application in different motion estimation situations. When the two algorithms work serially to yield ${\hat{X}}_{k+1}$ and ${\hat{Y}}_{k+1}$ successively, the entire algorithm complexity can be restricted to $O\left(n\right)$.
**Algorithm 1: SR-CICEKF**
**Input:** ${\tilde{\dot{X}}}_{k}$,${\hat{X}}_{k}$,$H_{k}$;1**While** *true* **do**2Update $F_{d}$,$Q_{d}$ according to $\tilde{\dot{X}}$ and ${\hat{X}}_{k}$; (←Propagation process in Section 3.1.1)3Update $P_{k+1|k}=F_{d}P_{k|k}F_{d}+Q_{d}$;4Update $K_{k}=P_{k+1|k}H_{k}^{T}{\left(H_{k}P_{k+1|k}H_{k}^{T}+R_{k}\right)}^{-1}$;5Calculate ${\tilde{z}}_{k}$ according to $H_{k}$; (←Measurement process in Section 3.1.2)6Update the current state ${\hat{X}}_{k+1|k+1}={\hat{X}}_{k+1|k}+{\hat{\tilde{X}}}_{k}$;7Update $P_{k+1|k+1}=(I_{27\times 27}-K_{k}H_{k})P_{k+1|k}{\left(I_{27\times 27}-K_{k}H_{k}\right)}^{T}+K_{k}R_{k}K_{k}^{T}$;8k=k+1;9**Output:** ${\hat{X}}_{k+1}$.
**Algorithm 2: MR-CICEKF**
**Input:** ${\tilde{\dot{Y}}}_{k}$,${\hat{Y}}_{k}$,$H_{k}^{r}$,X^k0,X^k1;1**While** *true* **do**2Update ${\hat{Y}}_{k}$ according to X^k0 and X^k1;3Update $F_{d}^{r}$,$Q_{d}^{r}$ according to ${\tilde{\dot{Y}}}_{k}$ and ${\hat{Y}}_{k}$; (←Propagation process in Section 3.2)4Update $P_{k+1|k}^{r}=F_{d}^{r}P_{k|k}^{r}F_{d}^{r}+Q_{d}^{r}$;5Update Kkr=Pk+1|krHkrHkrPk+1|krHkr+TRkr−1T;6Calculate ${\tilde{z}}_{rk}$ according to $H_{k}^{r}$; (←Measurement process in Section 3.2)7Update the current state ${\hat{Y}}_{k+1|k+1}={\hat{Y}}_{k+1|k}+{\hat{\tilde{Y}}}_{k}$;8Update $P_{k+1|k+1}^{r}=(I_{18\times 18}-K_{k}^{r}H_{k}^{r})P_{k+1|k}^{r}{\left(I_{18\times 18}-K_{k}^{r}H_{k}^{r}\right)}^{T}+K_{k}^{r}R_{k}^{r}K_{k}^{rT}$;9k=k+1;10**Output:** ${\hat{Y}}_{k+1}$.

When updating each current state, the quaternion error is recovered from the angular errors using the following equations [28]:

(38) δq^k≈12δθ^kq¯^k+1=1−δq^kδTq^k,δq^kTT if δq^kδTq^k≤1  11+δq^kδTq^k1,δq^kTT if δq^kδTq^k>1 .

## 4. Nonlinear Observability Analysis

### 4.1. Observability Analysis of the SR-CICEKF

When treating the filter as a nonlinear multi-input and multi-output system, its prerequisite for the filter to achieve correct estimation is that the system is observable. Researchers have proven that a filter possessing local weak observability can function properly, which is equivalent to the constructed observability matrix having full rank [21,34]. To simplify the discussion, a virtual measured angle velocity $\omega_{mic}$ and its bias $b_{\omega_{ic}}$ are assumed and partially coupled based on the IMU and visual measurement. Due to ${\bar{q}}_{ic}$ being in the filter state, and thus treated as paired with $\omega_{mic}$, this assumption does not affect the observability analysis. Thus, following Appendix A, the nonlinear measurement process of the SR-CICEKF can be expressed as follows:

(39) $$f\left(X\right)=\dot{X}=f_{0}+f_{1}\omega_{mi}+f_{2}\omega_{mic}+f_{3}a_{mi},$$

where for a general unit quaternion $\bar{q}$, there is $\Xi \left(\bar{q}\right)={\left[-q^{T}{\left(q_{0}I_{3\times 3}-\left\lfloor q\times \right\rfloor \right)}^{T}\right]}^{T}$ [21], and

(40) f0=μvvi+(1−μv)RcivcT 01×3 −Ribai−gT12Ξq¯icbωicT 12Ξq¯˙ibωiT 01×12Tf1=03×13 12Ξq¯iT 03×12Tf2=03×9 12Ξq¯icT03×16 Tf3=03×6 Ri03×20TT.

The measurement functions for the SR-CICEKF are designed as hX˙=h1,T…,h8TT, with $h_{1}=(p_{ic}-R_{ic}p_{i}^{c})\lambda$, $h_{2}={\bar{q}}_{ic}$, $h_{3}={\bar{q}}_{i}$, h4=q¯icq¯icT, h5=q¯iq¯iT, $h_{6}=v_{c}$, $h_{7}=\omega_{c}$, and $h_{8}=\omega_{i}$.

Following the Lie derivative rules in Appendix A and in [21], the observability matrix is as follows:

(41) $$\Omega =\left[\begin{array}{l} \nabla L^{0}h_{1}\\ \nabla L^{0}h_{2}\\ \nabla L^{0}h_{3}\\ \nabla L^{0}h_{4}\\ \nabla L^{0}h_{5}\\ \nabla L^{0}h_{6}\\ \nabla L^{0}h_{7}\\ \nabla L^{0}h_{8}\\ \nabla L_{f_{0}}^{1}h_{1}\\ \nabla L_{f_{0}}^{1}h_{3}\\ \nabla L_{f_{0}}^{2}h_{1} \end{array}\right]={\left[\begin{array}{ccccccccc} \overset{p_{ic}}{\overbrace{I_{3\times 3}}} & \overset{v_{c}}{\overbrace{0_{3\times 3}}} & \overset{v_{i}}{\overbrace{0_{3\times 3}}} & \overset{{\bar{q}}_{ic}}{\overbrace{G_{\left[1,4\right]}}} & \overset{{\bar{q}}_{i}}{\overbrace{0_{3\times 4}}} & \overset{\omega_{c}}{\overbrace{0_{3\times 3}}} & \overset{\omega_{i}}{\overbrace{0_{3\times 3}}} & \overset{b_{a}}{\overbrace{0_{3\times 3}}} & \overset{b_{\omega }}{\overbrace{0_{3\times 3}}}\\ 0_{4\times 3} & 0_{4\times 3} & 0_{4\times 3} & I_{4\times 4} & 0_{4\times 4} & 0_{4\times 3} & 0_{4\times 3} & 0_{4\times 3} & 0_{4\times 3}\\ 0_{4\times 3} & 0_{4\times 3} & 0_{4\times 3} & 0_{4\times 4} & I_{4\times 4} & 0_{4\times 3} & 0_{4\times 3} & 0_{4\times 3} & 0_{4\times 3}\\ 0_{1\times 3} & 0_{1\times 3} & 0_{1\times 3} & G_{\left[4,4\right]} & 0_{1\times 4} & 0_{1\times 3} & 0_{1\times 3} & 0_{1\times 3} & 0_{1\times 3}\\ 0_{1\times 3} & 0_{1\times 3} & 0_{1\times 3} & 0_{1\times 4} & G_{\left[5,4\right]} & 0_{1\times 3} & 0_{1\times 3} & 0_{1\times 3} & 0_{1\times 3}\\ 0_{3\times 3} & I_{3\times 3} & 0_{3\times 3} & 0_{3\times 4} & 0_{3\times 4} & 0_{3\times 3} & 0_{3\times 3} & 0_{3\times 3} & 0_{3\times 3}\\ 0_{3\times 3} & 0_{3\times 3} & 0_{3\times 3} & 0_{3\times 4} & 0_{3\times 4} & I_{3\times 3} & 0_{3\times 3} & 0_{3\times 3} & 0_{3\times 3}\\ 0_{3\times 3} & 0_{3\times 3} & 0_{3\times 3} & 0_{3\times 4} & 0_{3\times 4} & 0_{3\times 3} & I_{3\times 3} & 0_{3\times 3} & 0_{3\times 3}\\ 0_{3\times 3} & G_{\left[9,2\right]} & \mu_{v}I_{3\times 3} & G_{\left[9,4\right]} & 0_{3\times 4} & 0_{3\times 3} & 0_{3\times 3} & G_{\left[9,8\right]} & 0_{3\times 3}\\ 0_{4\times 3} & 0_{4\times 3} & 0_{4\times 3} & 0_{4\times 4} & 0_{4\times 4} & G_{\left[10,6\right]} & 0_{4\times 3} & 0_{4\times 3} & 0.5\Xi \left({\bar{q}}_{i}\right)\\ 0_{3\times 3} & 0_{3\times 3} & 0_{3\times 3} & G_{\left[11,4\right]} & G_{\left[11,5\right]} & 0_{3\times 3} & 0_{3\times 3} & -\mu_{v}R_{i} & G_{\left[11,9\right]} \end{array}\right]}_{31\times 29},$$

where the matrices G with the row and column indexes as subscripts do not contribute to the rank analysis.

To calculate the column rank, block Gaussian elimination can be applied. Once the corresponding block is an identity matrix, the relative column can be eliminated. Thus, only the last two columns are needed for the analysis and can be simplified as follows:

(42) $$\Omega^{\prime }=\left[\begin{array}{cc} 0 & 0.5\Xi \left({\dot{\bar{q}}}_{i}\right)\\ -\mu_{v}R_{i} & G_{\left[11,9\right]} \end{array}\right].$$

Therefore, if $\mu_{v}$ were non-zero, and any axis of the IMU was excited in any direction, $\Omega^{\prime }$ has full column rank, and Ω, thus, has full column rank. This means the SR-CICEKF has local weak observability [21,34]. The above conditions of achieving full rank are easily fulfilled during the application.

### 4.2. Observability Analysis of the MR-CICEKF

Resembling the previous analysis, the nonlinear system representing the measurement results of the MR-CICEKF can be expressed as follows:

(43) fY=Y˙=p˙r T03×3 03×3 q¯˙T 03×3 03×3T.

The measurement functions for MR-CICEKF are designed as hrY˙=hr1,T…,hr6TT, with $h_{r1}=p_{r}$, $h_{r2}={\bar{q}}_{r}$, $h_{r3}=v_{i0}$, $h_{r4}=v_{i1}$, $h_{r5}=\omega_{i0}$, and $h_{r6}=\omega_{i1}$.

After applying Lie derivative rules, the observability matrix of the MR-CICEKF system is constructed as follows:

(44) $$\Theta =\left[\begin{array}{l} \nabla L^{0}h_{r1}\\ \nabla L^{0}h_{r2}\\ \nabla L^{0}h_{r3}\\ \nabla L^{0}h_{r4}\\ \nabla L^{0}h_{r5}\\ \nabla L^{0}h_{r6} \end{array}\right]={\left[\begin{array}{cccccc} \overset{p_{r}}{\overbrace{I_{3\times 3}}} & \overset{v_{i0}}{\overbrace{0_{3\times 3}}} & \overset{v_{i1}}{\overbrace{0_{3\times 3}}} & \overset{{\bar{q}}_{r}}{\overbrace{0_{3\times 4}}} & \overset{\omega_{i0}}{\overbrace{0_{3\times 3}}} & \overset{\omega_{i1}}{\overbrace{0_{3\times 3}}}\\ 0_{4\times 3} & 0_{4\times 3} & 0_{4\times 3} & I_{4\times 4} & 0_{4\times 3} & 0_{4\times 3}\\ 0_{3\times 3} & I_{3\times 3} & 0_{3\times 3} & 0_{3\times 4} & 0_{3\times 3} & 0_{3\times 3}\\ 0_{3\times 3} & 0_{3\times 3} & I_{3\times 3} & 0_{3\times 4} & 0_{3\times 3} & 0_{3\times 3}\\ 0_{3\times 3} & 0_{3\times 3} & 0_{3\times 3} & 0_{3\times 4} & I_{3\times 3} & 0_{3\times 3}\\ 0_{3\times 3} & 0_{3\times 3} & 0_{3\times 3} & 0_{3\times 4} & 0_{3\times 3} & I_{3\times 3} \end{array}\right]}_{19\times 19}.$$

All the block columns in Θ have full column rank when the single-robot measurement yields non-zero outcomes, which means that the system has local weak observability [21,34]. 

## 5. Data Test

Two sets of motion simulations were performed to test the feasibility of the CICEKF algorithm. The motion curve was chosen as quintic, which is convenient for calculating the linear accelerations.

### 5.1. SR-CICEKF Simulation Test

To simplify the simulation for an individual sensor suite, the coordinate frames of the IMU and the stereo vision are assumed to coincide, and the biases are omitted. Once the parameters of the quintic curve are confirmed, the position coordinates of the curve can be treated as the position ground truth. By analyzing the projections of the curve on the three standard planes, the roll, pitch, and yaw orientations of the adjacent points can be achieved by allocating the starting and ending points, which yields the ground truth of the orientation. Thus, the ideal linear acceleration, linear velocity, and angular velocity data can be differentiated from the position and orientations, which are utilized as the filter input after being corrupted with Gaussian noise. 

**Figure 3:**
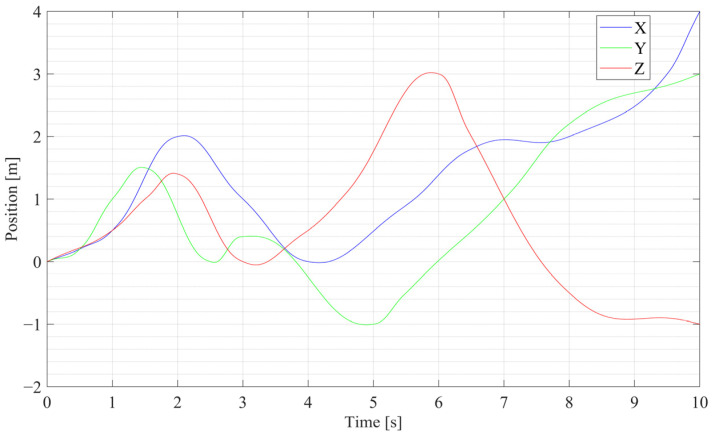
Position curves of the generated quintic trajectory.

**Figure 4:**
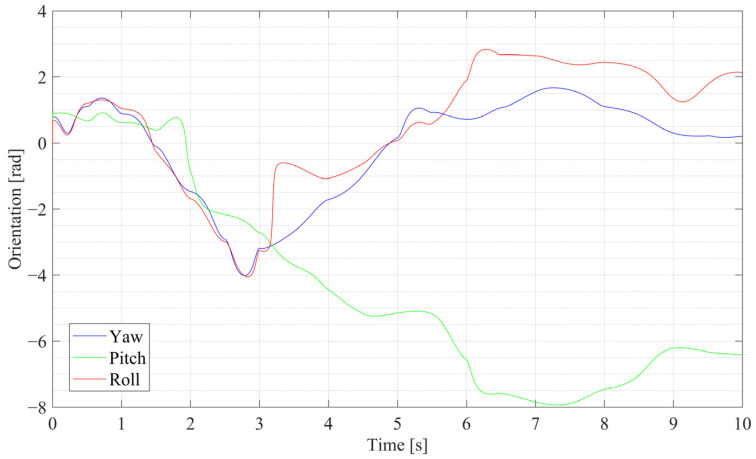
Orientation curves of the generated quintic trajectory.

The position and orientation curves of the generated quintic trajectory across all three axes are shown in Figure 3 and Figure 4, respectively. It should be noted that the change in the trajectory is designed in a short time span of 10 s with a 1000 Hz update rate, which means the change in simulation data is quite fast; for example, the maximum angular velocity is over 6 rad/s, and the max linear acceleration is over 11 m^2^/s.

$P_{k|k}$ can be asymptotically stable after a complete run with the randomized initial values. By reusing the stabilized $P_{k|k}$ in other runs, the initial convergence of the filter process can be greatly boosted. $R_{k}$ is designed as a skew-symmetric matrix containing small random values. The coefficients are set as $\mu_{v}=0.5$ and $\mu_{\omega }=0.5$. The initial position and orientation are set as [0, 0, 0]. 

**Figure 5:**
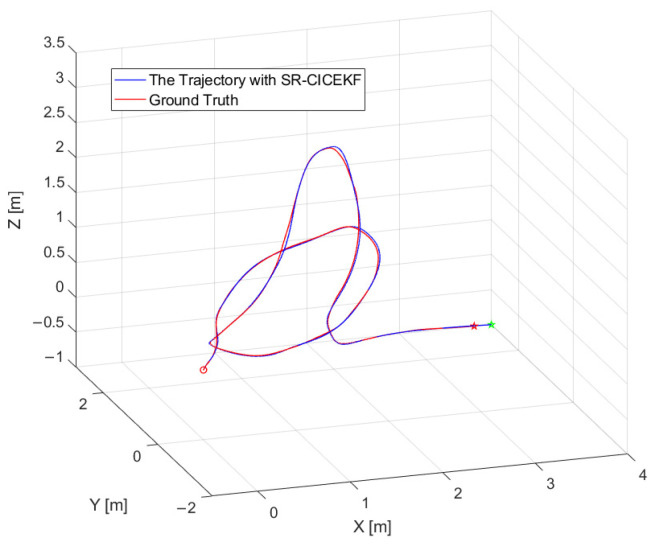
Three-dimensional position curves of the SR-CICEKF simulation.

**Figure 6:**
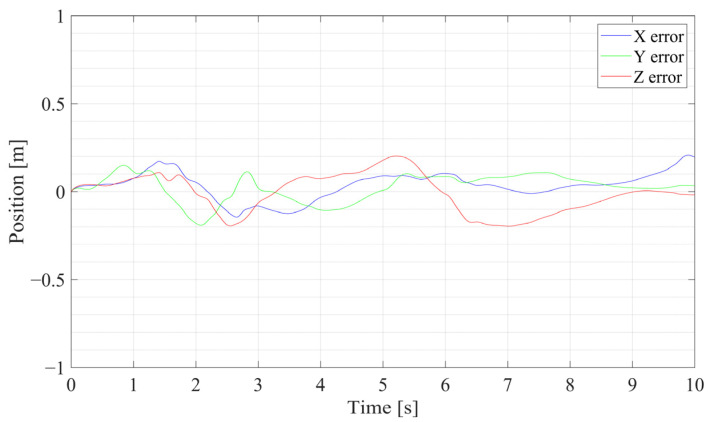
Position error curves of the SR-CICEKF simulation.

**Figure 7:**
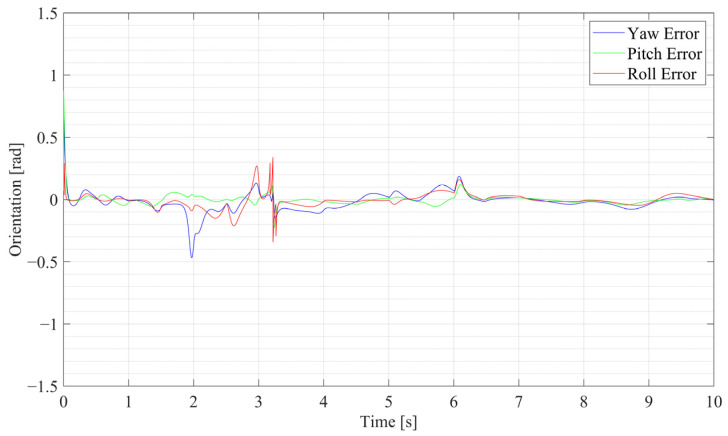
Orientation error curves of the SR-CICEKF simulation.

The three-dimensional position curves of the SR-CICEKF simulation are shown in Figure 5, with the circle mark as the starting point and the star markers as the ending points. The errors between the virtual measurement values and the filtered values of the position and orientation are shown in Figure 6 and Figure 7, respectively. The statistical results of RMSE, mean errors, and STD of the norm errors involving the three axes of the position and the orientation are listed in Table 2. 

By inspecting the curves and the statistical values, it can be concluded that the SR-CICEKF quickly converges under severe data changes, while it does not need careful parameter tuning for the first estimation of $P_{k|k}$. This indicates that the filter is robust, and the observability of the fusion process is identified indirectly.

### 5.2. MR-CICEKF Simulation

The goal of the simulation is to estimate the relative translation and rotation between the master and slave robots. The MR-CICEKF simulation utilizes the same quintic data as the target trajectories of both the master and slave robots. The absolute relative translation of the two robots is set as [1, 1, 1], and the relative rotation is set as [0, 0, 0]. The MR-CICEKF is tested independently of the SR-CICEKF. Thus, the performance of the MR-CICEKF is not affected by the possible inaccuracy produced by the SR-CICEKF process. The configurations of $P_{k|k}^{r}$ and $R_{k}^{r}$ are similar to the SR-CICEKF simulation, and the simulation input is corrupted with Gaussian noise.

**Figure 8:**
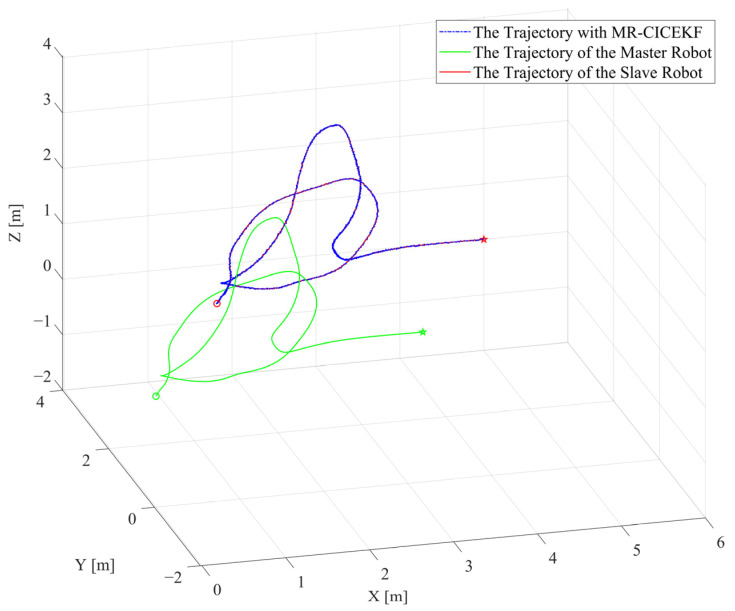
Three-dimensional position curves of the MR-CICEKF simulation.

Figure 8 depicts the three-dimensional position curves of the MR-CICEKF simulation, containing the ground truth of the master and slave robots’ trajectories, and the filtered slave robot trajectory, which is achieved by adding the estimated $p_{r}$ and ${\bar{q}}_{r}$ to the master trajectory. The circle marks are the starting points, and the star markers are the ending points. The statistical values of RMSE, mean errors, and STD of the norm errors involving the three axes of the position and the orientation obtained after comparing the ground truth with the filtered slave robot trajectories are listed in Table 3. 

The statistical values indicate that the final error is similar to the small white noise added. Thus, it can be concluded that the MR-CICEKF possesses good estimation accuracy, while the main error is caused by eliminating high-order terms during the state transition. This feature can significantly simplify further application since the MR-CICEKF contributes little error to the entire CICEKF process.

### 5.3. Dataset Test

To further analyze the performance of the CICEKF in real-world scenarios, a test was performed with the EuRoC dataset. The SR-CICEKF part is tested solely since the MR-CICEKF already showed great performance. The state-of-the-art visual SLAM method, ORB-SLAM V3 [16], was utilized as the visual black box measurement algorithm. The SR-CICEKF runs at 200 Hz, which is synchronous with the IMU update rate of the dataset. By employing EVO tools [35], the RPE features of the trajectories can be directly digitized. 

**Figure 9:**
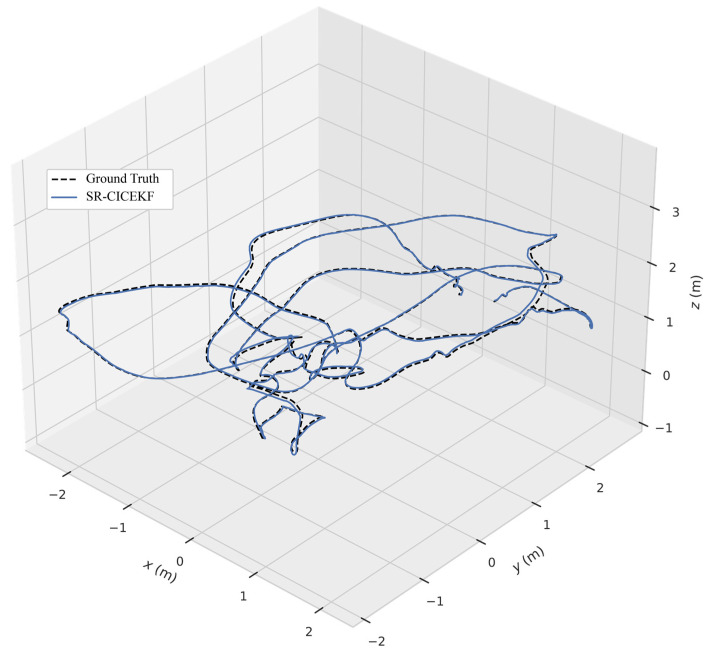
Comparison of the estimation trajectories of the SR-CICEKF and the ground truth.

**Figure 10:**
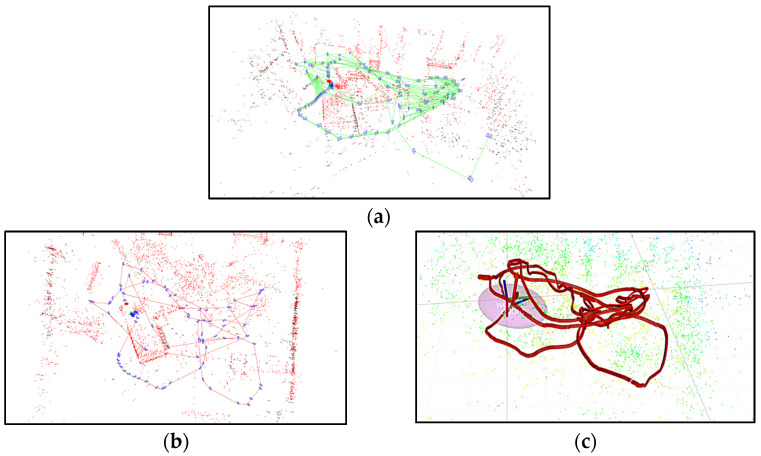
Dataset test scenarios: (**a**) stereo ORB-SLAM V3, (**b**) stereo ORB-SLAM V3 with the IMU, and (**c**) stereo MSCKF.

The stereo vision of ORB-SLAM V3 runs at 20 Hz, and all measurement frames were treated as keyframes. The coefficients were set as $\mu_{v}=0.9$ and $\mu_{\omega }=0.5$. The initial covariance matrix $P_{k|k}$ was tuned following the aforementioned strategy. The SR-CICEKF, stereo ORB-SLAM V3, and its variant, along with the stereo MSCKF [17], were carried out with the room dataset of EuRoC under the same computer configuration. Figure 9 shows the comparison of the estimation trajectories of the SR-CICEKF and the ground truth. Figure 10 shows the dataset test scenarios of the algorithms separately. The RPE comparison results between each algorithm with the ground truth achieved by utilizing the EVO tools are listed in Table 4. 

From the comparison, it can be concluded that by fusing IMU information and the pure visual black box measurement with the proposed SR-CICEKF algorithm, the accuracy has been greatly improved. Although the accuracy of the approach does not reach that of the optimization-based method, the fusion computational complexity is as low as $O\left(n\right)$. This can benefit the fusion with other visual measurement algorithms to improve the performance of trajectory estimation without affecting the computing efficiency. Furthermore, it can be combined with MR-CICEKF for the simultaneous estimation of multiple agents’ trajectories and collaborative configurations.

## 6. Experiments

**Figure 11:**
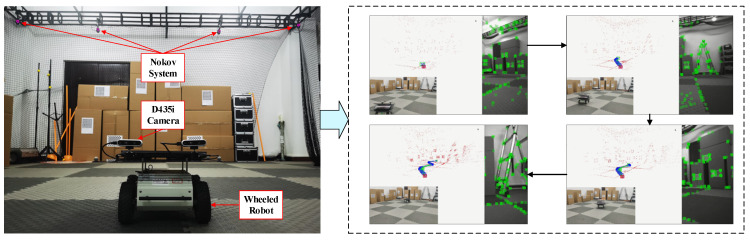
Experimental scenarios.

Two experiments were designed to focus the discussion on the feasibility of the proposed method. Firstly, the Intel RealSense D435i, which contains a stereo set of cameras and an IMU, was mounted on a wheeled robot that performs locomotion. The SR-CICEKF estimated the locomotive trajectory while the ground truth was measured by the NOKOV motion capture system. Then, the D435i camera data were utilized for collaborative loco-motion estimation. The experimental scenarios (Supplementary Materials) are shown in Figure 11.

For both parts of the CICEKF, the black-box stereo visual algorithm is ORB-SLAM V3 operating at 30 Hz, while the IMU update rate is set to 200 Hz. The calibration of the cameras and IMUs was conducted using Kalibr tools [36] to obtain the intrinsic and extrinsic parameters and the fixed configuration of the integrated sensors.

**Figure 12:**
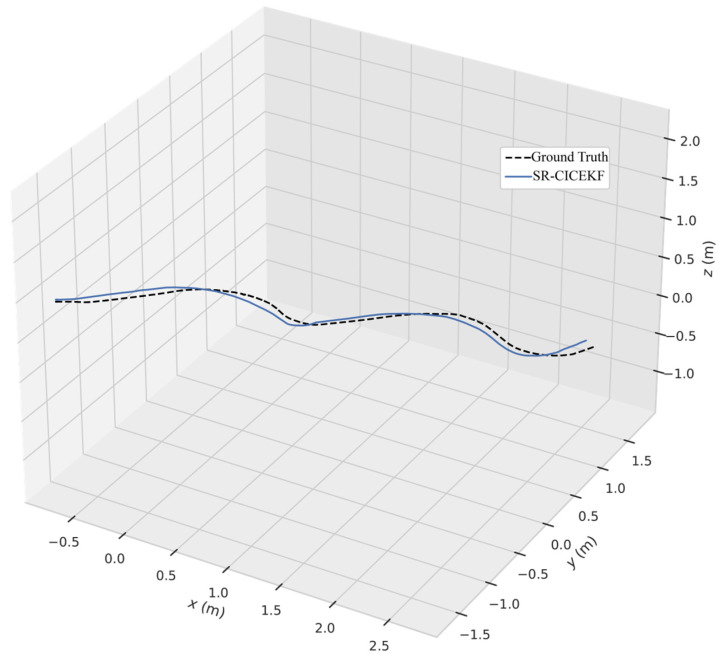
Comparison of the estimated trajectory and the ground truth of the SR-CICEKF.

During the experiment, we noticed that ORB-SLAM V3 and its IMU variant might not be stable under our experimental conditions due to the calibration accuracy and the IMU lacking sufficient excitation, since we used a slow ground vehicle as the motion platform. Therefore, to demonstrate the performance of the SR-CICEKF, the estimated trajectory was compared with that of stereo ORB-SLAM V3 and stereo MSCKF. The coefficients were set as $\mu_{v}=0.5$ and $\mu_{\omega }=0.5$. The comparison of the measured trajectory and the ground truth is shown in Figure 12. The RPE comparison results between each algorithm with the ground truth in a real scenario are listed in Table 5.

For the MR-CICEKF experiment, the two D435i cameras were calibrated with Kalibr tools to achieve a fixed relative configuration as both the ground truth and the initial guess of the filter. The relative configuration estimation of the MR-CICEKF was compared with the ground truth. The norm errors of the relative configuration estimation are listed in Table 6.

From the experimental results, it can be concluded that the proposed algorithm can not only improve the accuracy of pure visual measurement algorithms but also reliably estimate the relative configuration of multiple robots. 

## 7. Conclusions

This study introduces a novel motion estimation approach with a loosely coupled and dual-step structure for the autonomous and collaborative localization of multiple locomotive robots. Both steps of the algorithm can run standalone, serially, or in parallel while maintaining a computational complexity of $O\left(n\right)$, which allows for convenient improvement to pure vision measurement techniques after introducing an IMU. Both the simulations and experiments demonstrate that fusing data on the velocity level without post-optimization greatly contributes to the robot trajectory estimation accuracy. Due to current experimental limitations, further research will be focused on systematically examining how the coupling coefficients affect filter performance and stability across various integrated sensors and high-speed motion units.

## Figures and Tables

**Table 1:** Coordinate frames and notations.

Symbol	Description
*w*	Coordinate frame of the fixed world
*i*	Coordinate frame attached to the IMU’s rigid body
*c*	Coordinate frame attached to the rigid body of the dual-camera stereo vision
*ic*	Coordinate frame attached to the virtual rigid body of the IMU-aided camera system
$x_{A}^{B}$	x represents a general viable vector, A and B are the reference coordinate frames, e.g., $p_{A}^{B}$ denotes the linear translation in the coordinate frame B measured with respect to the coordinate frame A
xCD	x represents a general viable vector, C and D are the robot indexes, e.g., pCD denotes the linear translation of the D-th robot measured with respect to the C-th robot, particularly, xC=xCC.
$\left\lfloor x\times \right\rfloor$	Skew-symmetric matrix of x, and $\left\lfloor x\times \right\rfloor y=-x\left\lfloor y\times \right\rfloor$ [28] holds
p	The linear translation vector of rigid bodies along 3 axes, of which the quasi-quaternion description is $\bar{p}={\left[0,p^{T}\right]}^{T}$
$\bar{q}$	The unit quaternion following the Hamilton notation [21], written as $\bar{q}={\left[q_{0},q_{1},q_{2},q_{3}\right]}^{T}={\left[q_{0},q^{T}\right]}^{T}$
${\bar{q}}^{*}$	The conjugate form of $\bar{q}$, and ${\bar{q}}^{*}⊗\bar{q}=1$ holds
b	The uncertain bias of the measurement result
R	The equivalent rotation matrix of the quaternion $\bar{q}$, e.g., $R_{w}^{i}=R\left({\bar{q}}_{w}^{i}\right)$
n	White Gaussian noise vector with zero mean and covariance $\sigma^{2}$
$\dot{x}$,$\hat{x}$	The first-order time derivative form and the estimated form of the vector x, respectively
$\tilde{x}$	The error form of the vector x, which is defined as $\tilde{x}=x-\hat{x}$
$\delta \bar{q}$	The error of the quaternion $\bar{q}$, and $\delta \bar{q}={\hat{\bar{q}}}^{*}⊗\bar{q}$ holds

**Table 2:** The norm errors between the ground truth and the SR-CICEKF simulation results.

Translation RMSE	Translation Mean Error	Translation STD	Orientation RMSE	Orientation Mean Error	Orientation STD
0.1593 m	0.0429 m	0.1535 m	0.106 rad	0.0187 rad	0.1044 rad

**Table 3:** The norm errors between the ground truth and the filtered slave robot trajectories.

Translation RMSE	Translation Mean Error	Translation STD	Orientation RMSE	Orientation Mean Error	Orientation STD
0.01872 m	5.286 × 10^−6^ m	0.0187 m	0.0016 rad	4.95 × 10^−7^ rad	0.0016 rad

**Table 4:** Norm errors of the translations between the ground truth and the results of the dataset tests.

	Translation RMSE	Translation Mean Error	Translation STD
SR-CICEKF	0.004211 m	0.003982 m	0.001371 m
Stereo ORB-SLAM V3	0.03844 m	0.03428 m	0.01739 m
Stereo ORB-SLAM V3 with the IMU	0.003236 m	0.002785 m	0.001648 m
Stereo MSCKF	0.0556 m	0.048994 m	0.02629 m

**Table 5:** Norm errors of the translations between the ground truth and the experimental results.

	Translation RMSE	Translation Mean Error	Translation STD
SR-CICEKF	0.00459 m	0.006448 m	0.004528 m
Stereo ORB-SLAM V3	0.01723 m	0.01275 m	0.01159 m
Stereo MSCKF	0.01745 m	0.01292 m	0.01173 m

**Table 6:** Norm errors of the relative configuration estimation.

Translation RMSE	Translation Mean Error	Translation STD	Orientation RMSE	Orientation Mean Error	Orientation STD
0.009398 m	0.0001 m	0.0094 m	0.032 rad	0.00602 rad	0.03145 rad

## Data Availability

The source code presented in this study is available upon request from the corresponding author.

## Supplementary Materials

The following supporting information can be downloaded at: https://www.mdpi.com/article/10.3390/s25103086/s1.

## Appendix A

Assuming that the rotation $\bar{q}$ is accomplished during a certain period, and the corresponding body angular velocity is ω, there is $\dot{\bar{q}}=\frac{1}{2}\bar{q}⊗\bar{\omega }$ with $\bar{\omega }=[0,\omega ]$ [28]. The μ-th power of $\bar{q}$ is written as ${\bar{q}}^{\mu }$, which is a unit quaternion that denotes scaling the rotation angle around the virtual axis with $\mu \in \left[0,1\right]$ [30], and there is $\frac{d}{dt}\left({\bar{q}}^{\mu }\right)=\frac{1}{2}{\bar{q}}^{\mu }⊗\left(\mu \bar{\omega }\right)$ [29].

Rotating a three-element vector p with a rotation $\bar{q}$ is written as $\bar{q}⊗\bar{p}⊗{\bar{q}}^{*}≙R\left(\bar{q}\right)p$ with $\bar{p}={\left[0,p^{T}\right]}^{T}$.

For general quaternions, the error between the measurement and the expectation is defined as $\delta \bar{q}$ from $\bar{q}=\hat{\bar{q}}⊗\delta \bar{q}$. The derivative form is $\delta \dot{\bar{q}}={\hat{\bar{q}}}^{*}⊗\left(\dot{\bar{q}}-\dot{\hat{\bar{q}}}⊗\delta \bar{q}\right)$ [23].

According to $R\left(\delta \bar{q}\right)\approx I_{3}+\left\lfloor \delta \theta \times \right\rfloor$ [28], there is $R\left(\bar{q}\right)=\hat{R}\left(\hat{\bar{q}}\right)R\left(\delta \bar{q}\right)\approx \hat{R}\left(\hat{\bar{q}}\right)\left(I_{3}+\left\lfloor \delta \theta \times \right\rfloor \right)$.

Assuming that there are two quaternions, $\bar{q}={\left[q_{0},q^{T}\right]}^{T}$ and $\bar{p}={\left[p_{0},p^{T}\right]}^{T}$, the quaternion multiplication can be written as follows [28]:

(A1) $$\bar{q}⊗\bar{p}=\left[\begin{array}{cc} q_{0} & -q^{T}\\ q & q_{0}I_{3}+\left\lfloor q\times \right\rfloor \end{array}\right]⊗\bar{p}=\left[\begin{array}{cc} p_{0} & -p^{T}\\ p & p_{0}I_{3}+\left\lfloor p\times \right\rfloor \end{array}\right]⊗\bar{q}.$$

The Lie derivative of the measurement function $h\left(x\right)$ with respect to the vector $f\left(x\right)$ is written as follows [21]:

(A2) $$L_{f}h\left(x\right)=\nabla_{f}h\left(x\right)=\frac{\partial h\left(x\right)}{\partial x}f\left(x\right).$$

The k-th-order Lie derivative of $h\left(x\right)$ with respect to $f\left(x\right)$ is written as follows:

(A3) $$L_{f}^{k}h\left(x\right)=\frac{\partial \left(L_{f}^{k-1}h\left(x\right)\right)}{\partial x}f\left(x\right).$$

In particular, the zeroth-order Lie derivative of $h\left(x\right)$ is the measurement function itself, i.e., $L^{0}h\left(x\right)≜h\left(x\right)$.
